# Supplementary material for: Dynamic medial parietal and hippocampal deactivations under DMT relate to sympathetic output and altered sense of time, space, and the self
Source: Imaging Neurosci (Camb). 2025 Apr 16;3:imag_a_00541. doi: 10.1162/imag_a_00541 (PMC12319971; doi:10.1162/imag_a_00541)
Supplement: Supplementary Material [file imag_a_00541-supp.pdf]

Supplementary Materials for:

"Dynamic medial parietal and hippocampal deactivations under DMT relate to sympathetic output and altered sense of time, space, and the self"

Lorenzo Pasquini[1]*, Alexander J. Simon[2], Courtney L. Gallen[1], Hannes Kettner[1,3], Leor Roseman[3,4], Adam Gazzaley[1, 5,6], Robin L. Carhart-Harris[1,3,5], Christopher Timmermann[3,7]*

Content:

- Supplementary Methods: 2
- Supplementary Analyses: 1
- Supplementary Figures: 9
- Supplementary Table: 1

Supplementary Methods:

Participant's safety

DMT is known for inducing profound alterations of consciousness which can increase anxiety. This can be exacerbated in the MRI scanner, due to loud noises induced by scanning and feelings of claustrophobia which may arise. To enhance psychological safety, we developed enhanced ear protection for participants by combining in-ear headphones which blocked external sounds, together with earmuffs. Furthermore, just prior to the beginning of each scan, participants underwent a "body-scan" exercise, which was guided by the researcher via a microphone that was fed to the

in-ear headphones, whilst participants lay in the magnet. Finally, after the scan was over (and after completing the visual analogue scales), participants were directed to a room with soothing lighting and music and were encouraged to talk about their experiences with a researcher who used the micro-phenomenological interview method (Petitmengin, 2006), as employed in a previous DMT study. This enabled participants to make sense of the often-vivid effects of DMT and served as a form of "integration" of the experience, a method commonly used in clinical practice with psychedelics to foster safety (Johnson et al., 2008).

Structural MRI

Imaging was performed on a 3 T MRI (Siemens Magnetom Verio syngo MR B17) using a 12-channel head coil at Imanova, London, UK. Anatomical images were acquired using the ADNI-GO (Alzheimer's Disease Neuroimaging Initiative, Grand Opportunity) recommended MPRAGE parameters (1 mm isotropic voxels, TR = 2300 ms, TE = 2.98 ms, 160 sagittal slices, 256 × 256 in-plane FOV, flip angle = 9 degrees, bandwidth = 240 Hz/pixel, GRAPPA acceleration = 2).

Supplementary Analyses:

Post-hoc power analysis

Given the small samples size of the study, a post-hoc power analysis was performed using the publicly available toolbox G*Power v3.1 (https://www.psychologie.hhu.de/arbeitsgruppen/allgemeine-psychologie-und-arbeitspsychologie/gpower) (Faul et al., 2007). A Cohen's d of 1.08 was estimated using the *meanEffectSize* package on MATLAB, reflecting the effect size when comparing mean

heart rate estimates under placebo and DMT for State 4. With a sample size of 14, the post-hoc power analysis comparing the difference between two dependent means yielded a power of 0.96, indicating that our study had an 96% chance of detecting a significant difference between the groups at the alpha level of 0.05.

Supplementary Figures and Tables

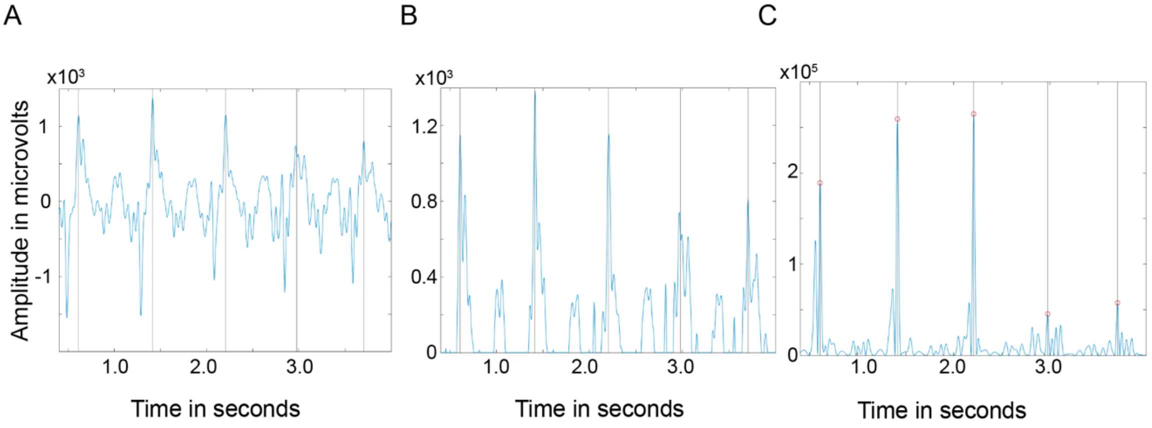

Supplementary Figure S1. EKG data preprocessing pipeline. (A) Four seconds of EKG data of a participant are shown for demonstrative purposes. **(B)** After visual inspecting the raw data, negative voltages were set to 0 to facilitate the detection of R peaks. **(C)** The positive voltage EKG was then processed via a wavelet transform, and then peaks were detected using the *findpeaks* function in MATLAB with manually adjusted minimum height and minimum inter-beat distance. Vertical gray lines indicate peaks identified through the semi-automatic algorithm in panel **C** and correspondent portions of data in panels **A** and **B**.

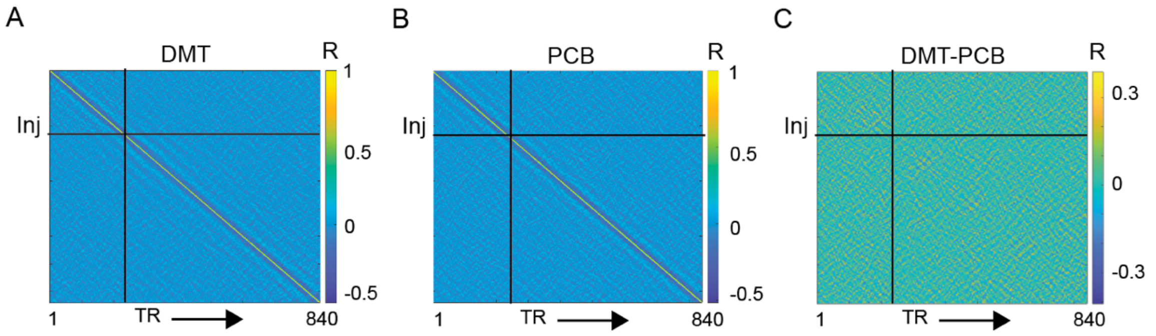

Supplementary Figure S2. Filtering of low frequencies removes dynamic patterns from rs-fMRI data. Removing frequencies below 0.01 Hz and linear detrending (as done in the parent paper Timmermann et al., 2023b) removes dynamic patterns from the rs-fMRI data. For this reason, only a low-pass filter removing frequencies >0.08 Hz and quadratic detrending were applied.

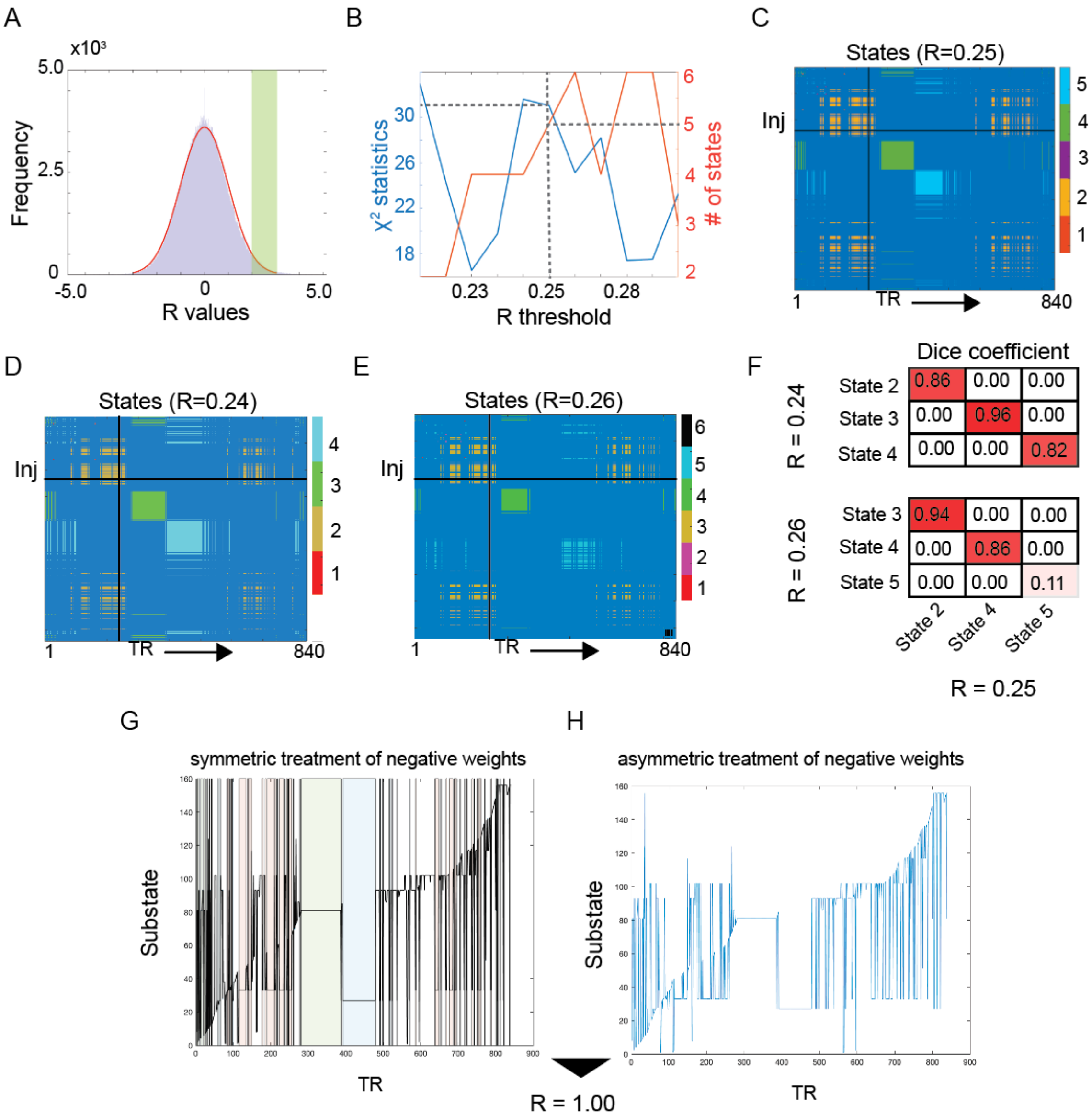

Supplementary Figure S3. Optimal threshold for brain activity substate identification. (A) Histogram of Pearson's correlation values extracted from the group-averaged subtraction matrix of time-resolved activity similarity. The light green frame indicates the range of Pearson's correlation values at the right of the positive distribution which were iteratively used to query the optimal value to be used to threshold the matrix (R = 0.20-0.30). **(B)** The group-averaged subtraction matrix of time-resolved activity similarity was thresholded for Pearson's correlation values ranging from R = 0.20-0.30, in

incremental steps of 0.01 (see x axis). Each of these thresholds yielded a distinct amount of brain activity substates (# of substates, in red on the right y axis) differentially occupied before and after the injection (indexed by mean X^2 statistics, in blue on the left y axis). The R threshold of 0.25 (vertical dotted line) was chosen since it maximized the number of brain activation substates separately occupied either during the pre- or post-injection periods (see horizontal dotted lines, five substates and X^2 = 31.1). Brain activity substates identified by thresholding the group-averaged subtraction matrix for Pearson's' correlation values of **(C)** 0.25 (five substates, X^2 = 31.1), **(D)** 0.24 (four substates, X^2 = 31.6), or **(E)** 0.26 (six substates, X^2 = 25.2). These two alternative thresholds were investigated since these also yielded a large number of brain activity substates differentially occupying either the pre- or post-injections periods. In the substate occupation matrices, only brain activity substates differentially occupying either the pre- or post-injection periods are shown. **(F)** A Dice coefficient was then used to quantify whether identified brain activity substates were found during overlapping periods of the scanning sessions when using a threshold of 0.25 versus thresholds of 0.24 or 0.26. **(G)** Community indexes identified through the Louvain algorithm using a threshold of 0.25 and symmetric treatment of negative weights. Shades in the background reflect periods of the scan corresponding to the primary brain activity substates described in the main findings, including State 2 (red shade), State 4 (green shade), and State 5 (cyan shade). **(H)** Identical findings were derived when using an asymmetric treatment of negative weights, one of the major hyperparameters that can be changed in the Louvain algorithm.

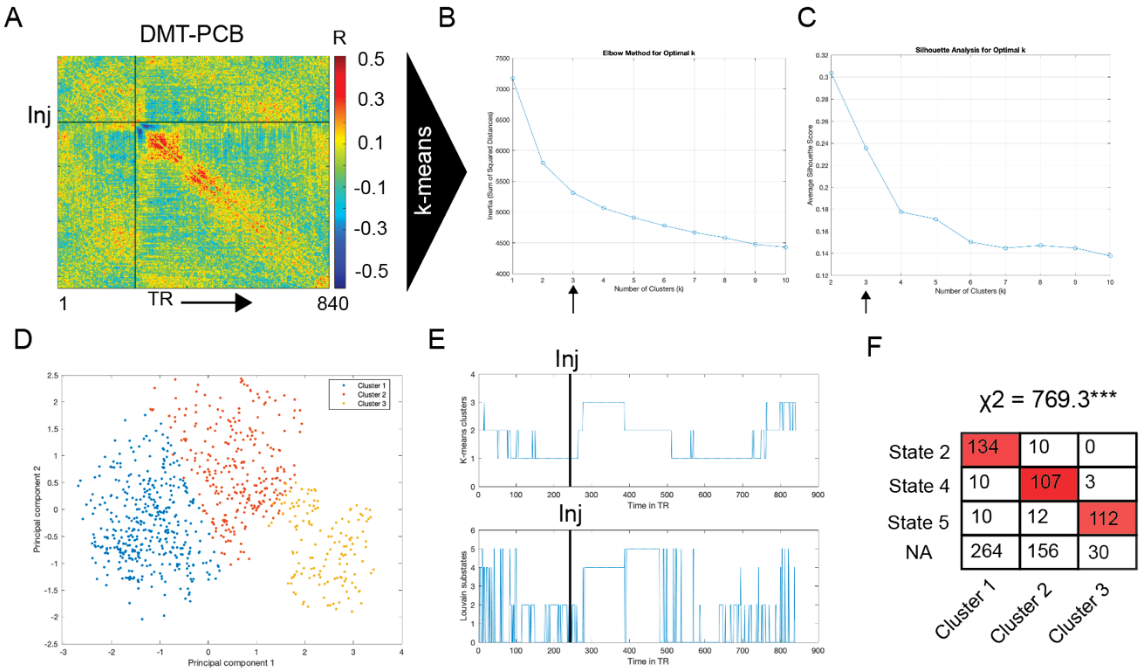

Supplementary Figure S4. K-means clustering. (**A**) K-means clustering (10,000 iteration, 10 replications) was applied to the mean subtraction matrix reflecting how continuous brain activity similarity varies across the DMT and placebo conditions. Several clustering solutions where explored, with elbow plots (**B**) and silhouette plots **(C)** supporting a clustering solution with three clusters **(D)**. **(E)** Time series showing how individual TRs were assigned to one of the three identified k-means clusters (top) or to the primary brain activity substates identified with the Louvain algorithm (bottom; States 2, 4, and 5). Black vertical line depicts time of injection (Inj). **(F)** Substantial overlap of States 2, 4, and 5 with k-means clusters 1,2, and 3, suggesting that both methods are able to identify similar brain activity substates. When using the Louvain algorithm, specific TRs remained unassigned since the identified substates were not occupied for substantial amounts of time. This suggest that the Louvain algorithm is more sensitive than the k-means algorithm at identifying discrete brain activity substates from the DMT – placebo subtraction matrix. ***p<0.005; X^2= Chi-square statistics; NA=not assigned.

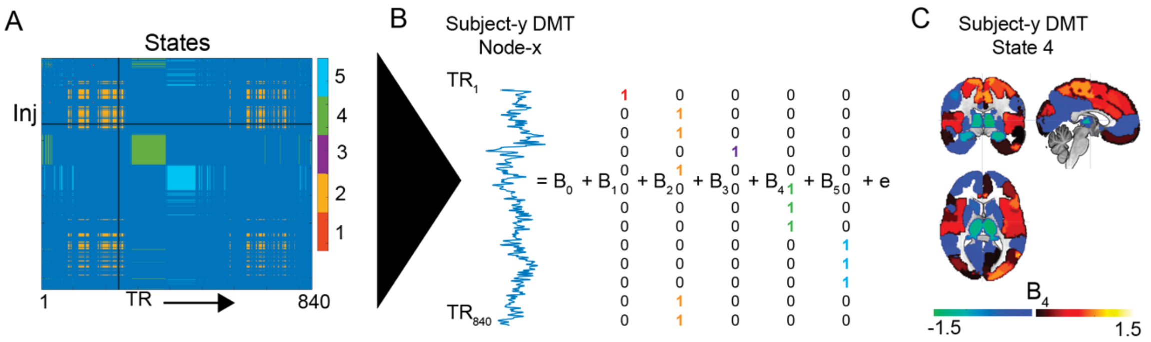

Supplementary Figure S5. Linear regression model used to derive subject specific brain activity substate maps. (**A**) The community detection algorithm applied on the group-averaged subtraction matrix of time-resolved activity similarity identified five brain activity substates differentiating the DMT from the placebo condition. This analysis resulted in a set of vectors reflecting the assignment of each time point to one of the five identified brain activity substates. (**B**) For each individual and for each condition (e.g., Subject-y under DMT), we derived the mean activity time series for each brain node (e.g., Node-x), which were used as dependent variables in a set of linear regression analyses, while the vectors reflecting community affiliation were entered as predictors. **(C)** This procedure was repeated for every node in the brain, resulting in individual estimates of brain substate activity maps (e.g., first post-injection State 4 of Subject-y under DMT).

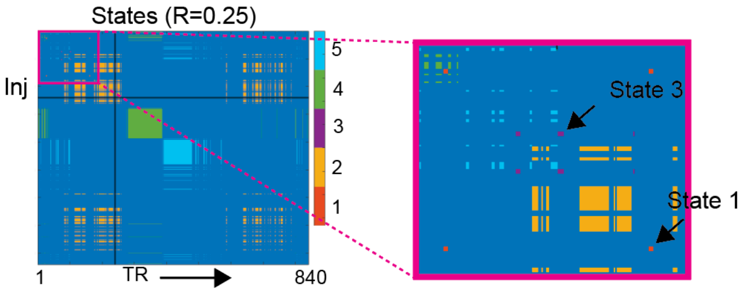

Supplementary Figure S6. Magnified detail of States 1 and 3. Magnified detail (pink square) showing the occurrence of State 1 (in red) and State 3 (in purple). Although both substates occupied the pre-injection period for a significant higher amount of time than the post-injection period, they also occupied the pre-injection period for only a brief amount of time (<5%) to warrant further investigation. Arrows depict examples of State 1 and 3 instances.

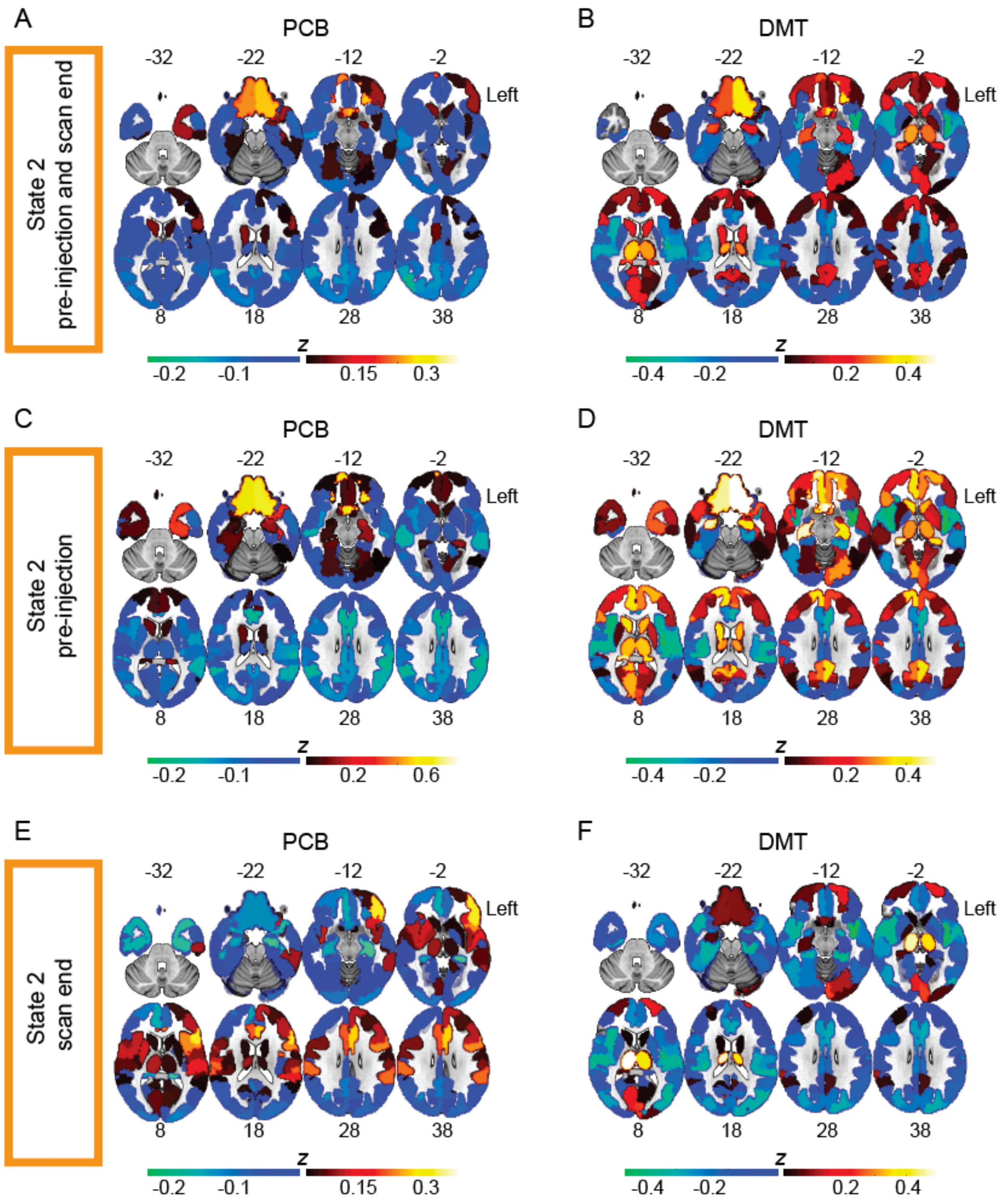

Supplementary Figure S7. Average State 2 activity average maps before and after the injection. Activity maps for State 2 averaged across instances of this state occurring before and after the injection of DMT once under placebo (PCB; **A)** and once under DMT **(B)**. Activity maps for State 2 averaged across instances of this state occurring before the injection of DMT once under placebo **(C)** and once under DMT **(D)**. Activity maps for State 2 averaged across instances of this state occurring after the injection of DMT once under

placebo **(E)** and once under DMT **(F)**. For the DMT condition, note consistent hyperactivity of subcortical, orbitofrontal, frontal, and medial parietal areas across pre- and post-injection periods (panels D and F).

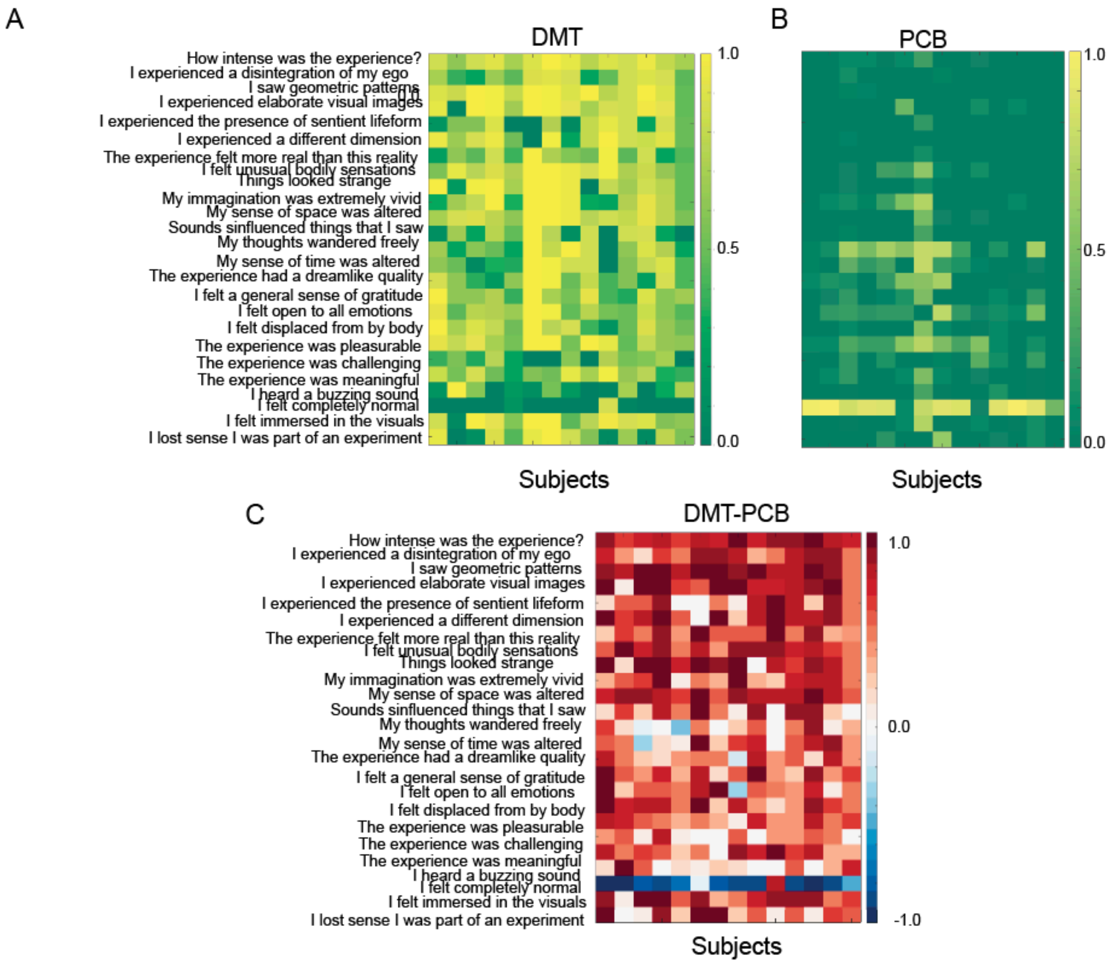

Supplementary Figure S8. Individual visual analog rating scales and DMT-placebo comparisons for selected ratings. Individual rating scores assessed through visual analog scales after the end of the scanning session (**A**) once under DMT (left) and (**B**) once under placebo (PCB, right). Warmer colors reflect higher scores. (**C**) Heatmap reflecting the difference in individual rating scores when comparing the DMT and the placebo conditions (DMT-PCB). Warmer colors reflect higher scores under DMT, colder colors reflect lower scores under DMT. All questionnaire scores where significantly increased under DMT when compared to placebo, with the exception of *"I felt completely normal",* which was significantly lower under DMT (p < 0.05 FDR corrected for multiple comparisons).

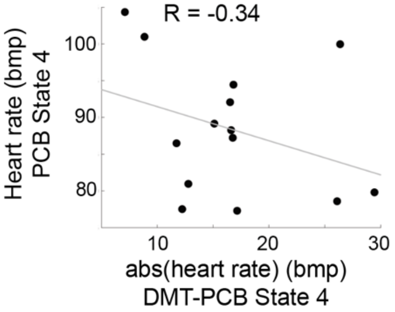

Supplementary Figure S9. State 4 absolute mean heart rate change and mean heart rate under placebo. Absolute mean heart rate change in State 4 did not significantly correlate with mean heart rate in State 4 under placebo.

	β DMT-order	SE	*t*	*p*
l-Hipp/MPC activity decreases in State 4	-0.09	0.21	-0.44	0.666
r-STL activity increases in State 4	0.82	0.38	2.13	*0.054*
Mean heart rate change in State 4	-0.81	8.34	-0.09	0.924

Supplementary Table S1. Order of DMT dosing day does not predict brain activity or mean heart rate changes in State 4. Three separate linear regression models were used to assess the relationship between order of DMT dosing (whether at the first or at the second visit) and changes in brain activity and mean heart rate during State 4, the first post-injection brain activity substate. Order of DMT administration did not significantly predict decreases in brain activity and heart rate, although it approached significance in predicting right superior temporal lobe activity increases under DMT. L-Hipp = left hippocampus; MPC = medial parietal cortex; r-STL = right superior temporal lobe
